# Fitness Level and Not Aging *per se*, Determines the Oxygen Uptake Kinetics Response

**DOI:** 10.3389/fphys.2018.00277

**Published:** 2018-03-29

**Authors:** Mitchell A. George, Kaitlin M. McLay, Patricia K. Doyle-Baker, Raylene A. Reimer, Juan M. Murias

**Affiliations:** ^1^Faculty of Kinesiology, University of Calgary, Calgary, AB, Canada; ^2^Faculty of Environmental Design, University of Calgary, Calgary, AB, Canada; ^3^Department of Biochemistry & Molecular Biology, Cumming School of Medicine, University of Calgary, Calgary, AB, Canada

**Keywords:** aging, training status, oxygen delivery, vascular responsiveness, near-infrared spectroscopy

## Abstract

Although aging has been associated to slower $\dot{\text{V}}$O_2_ kinetics, some evidence indicates that fitness status and not aging *per se* might modulate this response. The main goal of this study was to examine the $\dot{\text{V}}$O_2_, deoxygenated hemoglobin+myoglobin (deoxy-[Hb+Mb]) kinetics, and the NIRS-derived vascular reperfusion responses in older compared to young men of different training levels (i.e., inactive, recreationally active, and endurance trained). Ten young inactive [YI; 26 ± 5 yrs.; peak $\dot{\text{V}}$O_2_ ($\dot{\text{V}}$O_2peak_), 2.96 ± 0.55 L·min^−1^], 10 young recreationally active (YR; 26 ± 6 yrs.; 3.92 ± 0.33 L·min^−1^), 10 young endurance trained (YT; 30 ± 4 yrs.; 4.42 ± 0.32 L·min^−1^), 7 older inactive (OI; 69 ± 4 yrs.; 2.50 ± 0.31 L·min^−1^), 10 older recreationally active (OR; 69 ± 5 yrs.; 2.71 ± 0.42 L·min^−1^), and 10 older endurance trained (OT; 66 ± 3 yrs.; 3.20 ± 0.35 L·min^−1^) men completed transitions of moderate intensity cycling exercise (MODS) to determine $\dot{\text{V}}$O_2_ and deoxy-[Hb+Mb] kinetics, and the deoxy-[Hb+Mb]/$\dot{\text{V}}$O_2_ ratio. The time constant of $\dot{\text{V}}$O_2_ (τ$\dot{\text{V}}$O_2_) was greater in YI (38.8 ± 10.4 s) and OI (44.1 ± 10.8 s) compared with YR (26.8 ± 7.5 s) and OR (26.6 ± 6.5 s), as well as compared to YT (14.8 ± 3.4 s), and OT (17.7 ± 2.7 s) (*p* < 0.05). τ$\dot{\text{V}}$O_2_ was greater in YR and OR compared with YT and OT (*p* < 0.05). The deoxy-[Hb+Mb]/$\dot{\text{V}}$O_2_ ratio was greater in YI (1.23 ± 0.05) and OI (1.29 ± 0.08) compared with YR (1.11 ± 0.03) and OR (1.13 ± 0.06), as well as compared to YT (1.01 ± 0.03), and OT (1.06 ± 0.03) (*p* < 0.05). Similarly, the deoxy-[Hb+Mb]/ $\dot{\text{V}}$O_2_ ratio was greater in YR and OR compared with YT and OT (*p* < 0.05). There was a main effect of training (*p* = 0.033), whereby inactive (*p* = 0.018) and recreationally active men (*p* = 0.031) had significantly poorer vascular reperfusion than endurance trained men regardless of age. This study demonstrated not only that age-related slowing of $\dot{\text{V}}$O_2_ kinetics can be eliminated in endurance trained individuals, but also that inactive lifestyle negatively impacts the $\dot{\text{V}}$O_2_ kinetics response of young healthy individuals.

## Introduction

Studies assessing the physiological responses to exercise across the aging spectrum have demonstrated age-related declines within the O_2_ transport system, as determined by reductions in peak oxygen uptake ($\dot{\text{V}}$O_2peak_) (Astrand, 1976; Fitzgerald et al., 1997). Although endurance trained older individuals demonstrate a greater $\dot{\text{V}}$O_2peak_ compared to their untrained counterparts, an age-related decline in $\dot{\text{V}}$O_2peak_ is still inevitable (Wilson and Tanaka, 2000). Despite the prognostic values of $\dot{\text{V}}$O_2peak_ to predict a minimum level of fitness required to perform activities of daily living (ADL) without incurring fatigue that would negatively affect independent living, there are other measures of cardiovascular function including $\dot{\text{V}}$O_2_ kinetics and vascular responsiveness that are used to assess the ability of older individuals to perform ADLs. In fact, once a given functional threshold is achieved (i.e., a $\dot{\text{V}}$O_2peak_ of ~30 mL·kg^−1^·min^−1^) (Paterson et al., 2007), ADLs are performed at submaximal intensities of exercise, which makes measures of cardiovascular adjustments at these intensities very important.

To further understand age-related limitations within the path of O_2_ transport during submaximal exercise performed within the moderate intensity domain, researchers have investigated the mechanisms that control the dynamic adjustment of oxygen consumption ($\dot{\text{V}}$O_2_) during square-wave exercise transitions from a given baseline metabolic rate, to a higher metabolic demand below the gas exchange threshold (GET) (Babcock et al., 1994; DeLorey et al., 2007; duManoir et al., 2010; Murias et al., 2010; Grey et al., 2015). Using this model, an instantaneous increase in metabolic demand [typically power output (PO) during cycling exercise] produces a mono-exponential increase in the adjustment of oxidative phosphorylation [represented by the phase II $\dot{\text{V}}$O_2_ time constant (τ$\dot{\text{V}}$O_2_)]. The oxygen deficit generated by this mono-exponential increase in $\dot{\text{V}}$O_2_ represents an increase in metabolic demand that is partly sustained by non-oxidative sources until a steady state in $\dot{\text{V}}$O_2_ is achieved. Previous research has typically shown a slower rate of adjustment of oxidative phosphorylation ($\dot{\text{V}}$O_2_ kinetics) in older compared to young individuals (Bell et al., 2001; Grassi, 2001; Hughson et al., 2001; duManoir et al., 2010; Murias et al., 2014; McLay et al., 2017). Despite this observation, a faster $\dot{\text{V}}$O_2_ kinetics response can be observed in older individuals subsequent to endurance exercise training interventions (Babcock et al., 1994; Bell et al., 2001; Murias et al., 2010, 2016; Keir et al., 2016; McLay et al., 2017). This observation demonstrates that this population is still malleable to produce adaptations within the cardiovascular system and within the skeletal muscle (Belman and Gaesser, 1991) that would result in a faster adjustment of $\dot{\text{V}}$O_2_ at the onset of exercise. Indeed, a recent study in a population of chronically endurance trained older individuals demonstrated $\dot{\text{V}}$O_2_ kinetics responses in these individuals that were as fast as those observed in younger counterparts (Grey et al., 2015), prompting the authors to suggest that fitness level and not aging might be the main factor determining the dynamic adjustment of the $\dot{\text{V}}$O_2_ response during the exercise on-transient (Grey et al., 2015).

Interestingly, this previous study (Grey et al., 2015) not only indicated that endurance trained older individuals had the same $\dot{\text{V}}$O_2_ kinetics as those observed in young endurance trained participants, but also that recreationally active young and middle-aged individuals showed slower $\dot{\text{V}}$O_2_ kinetics as compared to their endurance trained counterparts. These data re-enforce the idea that even young individuals might have a relatively slow rate of adjustment of oxidative phosphorylation when they are not in the upper end of the fitness level continuum. Unfortunately, the study of Grey et al. (2015) did not consider evaluating sedentary individuals. If fitness level and not aging *per se* is a key factor modulating the dynamic adjustment of $\dot{\text{V}}$O_2_, then not only chronically endurance trained older individuals should demonstrate a $\dot{\text{V}}$O_2_ kinetics that is as fast as that observed in their younger counterparts, but also young sedentary individuals should demonstrate a $\dot{\text{V}}$O_2_ kinetics response that is as slow as that observed in their older counterparts.

Therefore, the main goal of this study was to examine the $\dot{\text{V}}$O_2_ kinetics response in older compared to young individuals of different training levels (i.e., inactive, recreationally active, and endurance trained). It was hypothesized that there would be a continuous increase in the τ$\dot{\text{V}}$O_2_ from endurance trained to recreationally active to sedentary men, in both the young and older groups, so that fitness level would be a key modulator of the $\dot{\text{V}}$O_2_ kinetics response independently of age.

## Materials and methods

### Participants

Fifty-seven men were recruited from community associations across the city of Calgary, online community groups, and through word of mouth. All participants were healthy, non-obese, non-smokers who were not taking any medications that would affect their hemodynamic responses to exercise. Participants were separated into two groups, young (18–45 years) and older (60–75 years). Each group was further separated into three categories: inactive, recreationally active and endurance trained yielding six groups: young inactive (YI; *n* = 10), young recreationally active (YR; *n* = 10), young endurance trained (YT; *n* = 10), older inactive (OI; *n* = 7), older recreationally active (OR; *n* = 10), and older endurance trained (OT; *n* = 10). This study was carried out in accordance with the recommendations of the University of Calgary Conjoint Health Research Ethics Board with written informed consent from all participants. All participants gave written informed consent in accordance with the Declaration of Helsinki. The protocol was approved by the University of Calgary Conjoint Health Research Ethics Board (REB16-0578).

### Participant fitness level

The inactive men were engaged only in very light activity such as walking. The recreationally-trained men were regularly active (2–4 times per week), engaging in a mix of resistance and endurance exercise, as well as recreational sporting activities (i.e., exercising/playing sports for health). The endurance-trained men were competitive and/or active endurance athletes. All endurance athletes had been engaging in activity ≥4 times per week, with a mix of endurance and high-intensity exercise (i.e., exercising for competition) for at least the last 4 consecutive years.

### Experimental design

Participants were invited to the Laboratory to complete the experimental procedures on 3 separate days. All protocols were conducted in an environmentally-controlled laboratory (i.e., temperature ~21°C, relative humidity ~35%). Participants were instructed to refrain from vigorous physical activity, caffeine and alcohol for at least 12 h prior to each testing session.

On the first visit, participants performed a ramp incremental exercise test to exhaustion (50 W baseline for 4 min followed by a 15 W min^−1^ ramp for OU, 25 W min^−1^ ramp for YU, YR, OR, and OT and a 30 W min^−1^ ramp for YT) on a magnetically braked cycle ergometer (Velotron RacerMate Inc., Seattle, WA, USA) for determination of $\dot{\text{V}}$O_2peak_, peak power output (PO_peak_), and the GET. The test was terminated once participants were unable to continue pedaling at a constant cadence despite strong verbal encouragement. Participants only received external motivation near the end of the test to promote maximal effort. The $\dot{\text{V}}$O_2peak_ was defined as the greatest 20 s $\dot{\text{V}}$O_2_ ($\dot{\text{V}}$O_2_) computed from a rolling average, and PO_peak_ was defined as the PO achieved at termination of the ramp incremental test. The GET was determined by visual inspection and defined as the $\dot{\text{V}}$O_2_ at which the rate of CO_2_ output ($\dot{\text{V}}$CO_2_) began to increase out of proportion in relation to $\dot{\text{V}}$O_2_, with a systemic rise in minute ventilation ($\dot{\text{V}}$E) in relation to $\dot{\text{V}}$O_2_ and in end-tidal PO_2_, whereas the ventilatory equivalent of $\dot{\text{V}}$CO_2_ ($\dot{\text{V}}$E/$\dot{\text{V}}$CO_2_) and end-tidal PCO_2_ was stable (Beaver et al., 1986).

The second visit was separated by at least 48 h from the first one and participants underwent a vascular occlusion test to examine vascular responsiveness (described in detail below).

On the third day, participants performed a cycling test consisting of step transitions in PO from a 20 W baseline to a moderate-intensity, constant load PO that elicited a $\dot{\text{V}}$O_2_ corresponding to 80% of the GET. Each participant performed three repetitions of this step transition consisting of 6 min of pedaling at 20 W followed by 6 min of pedaling at a PO corresponding to 80% GET. Repeated transitions were performed to increase confidence in the parameter estimation as indicated by Lamarra et al. (1987) and Rossiter et al. (2000) and as experimentally recommended by Spencer et al. (2011).

### Cycling test measurements

Breath-by-breath gas exchange and ventilation, were continuously measured using a metabolic cart (Quark CPET, COSMED, Rome, Italy), as previously described in our laboratory (Mattioni Maturana et al., 2017). Calibration occurred before each test as recommended by the manufacturer.

Local muscle deoxygenation (deoxy-[Hb+Mb]) of the quadriceps vastus lateralis muscle was measured using a frequency-domain multi-distance near-infrared spectroscopy (NIRS) system (OxiplexTS, Model 92505; ISS, Champaign, IL, USA) as described elsewhere (Spencer et al., 2012). Briefly the NIRS probe was secured using a tightened black elastic strap (to mitigate movement) and covered with an optically dense, black vinyl sheet to minimize the intrusion of extraneous light. An elastic tensor bandage was then loosely wrapped around the site, to avoid constricting blood flow, but to further minimize movement and light intrusion. The probe stayed attached to the participant for the duration of testing. The NIRS system was composed of a single channel consisting of eight laser diodes operating at two wavelengths (λ = 690 and 828 nm; four at each wavelength) that were pulsed in a rapid succession down a photomultiplier tube. A rigid plastic NIRS probe (connected to laser diodes and photomultiplier tube by optical fibers) consisted of two parallel rows of light-emitter fibers and one detector fiber bundle; the source–detector separations for this probe were 2.0, 2.5, 3.0, and 3.5 cm for both wavelengths. The probe was placed on the belly of the muscle, at the distal end of the vastus lateralis muscle. The NIRS measurements were collected continuously for the entire duration of each test.

The near-infrared spectrometer was calibrated at the beginning of each testing session after a warm-up period of at least 30 min. The calibration was performed with the probe placed on a calibration block (phantom) with absorption (μA) and reduced scattering coefficients (μs′) previously measured; thus, correction factors were determined and were automatically implemented by the manufacturer's software for the calculation of the μA and μs′ for each wavelength during the data collection. Calculation of deoxy-[Hb+Mb] reflected continuous measurements of μs′ made throughout each testing session (i.e., constant scattering value was not assumed). Data were stored online at an output frequency of 2 Hz but were reduced to 1 s bins for all subsequent analyses within the present study.

### Vascular occlusion test

Participants laid supine on a plinth and following a 10-min rest-period, the NIRS probe was placed on the muscle belly of the tibialis anterior. A pneumatic cuff connected to an automatic rapid inflation system (Hokanson E20 AG101, Bellevue, WA, USA) was used for occlusion of blood flow, and placed below the knee (~5 cm distal to the popliteal fossa). Occlusion pressure was set to 250 mm Hg for the occlusion time. NIRS measurements were collected continuously at an output frequency of 2 Hz for the entire duration of each test (5 min of baseline, 5 min of occlusion, and 8 min following cuff release) (McLay et al., 2016a).

### Data analysis

$\dot{\text{V}}$O_2_ data were edited on an individual basis by removing aberrant data points that laid 3 *SD* outside of the local mean. During moderate intensity exercise (MOD) transitions, data for each repetition of a similar protocol were then linearly interpolated to 1 s intervals, time-aligned such that time zero represented the onset of the transition, and ensemble-averaged to yield a single averaged response for each participant for a given exercise protocol. These averaged responses were further time-averaged into 5 s bins. The on-transient responses for $\dot{\text{V}}$O_2_ were modeled using the following equation:

(1) $$\begin{array}{r} {\text{Y}}_{\left(\text{t}\right)}={\text{Y}}_{\text{bsln}}+\text{Amp }\left(1-\;{\text{e}}^{-\frac{\left(\text{t}-\text{TD}\right)}{\text{τ}}}\right) \end{array}$$

where Y_(t)_ represents the $\dot{\text{V}}$O_2_ at any given time (t); Y_bsln_ is the steady state baseline value of Y before an increase in WR; Amp is the amplitude of the increase in Y above Y_bsln_; and TD is the time delay (such that the model is not constrained to pass through the origin); τ is the time constant of the response (or the time required to attain 63% of the steady-state amplitude). The first 22 s of the $\dot{\text{V}}$O_2_ data were not included in the fitting of the phase II $\dot{\text{V}}$O_2_ response to minimize the effect of the phase I response in younger individuals (27 s for older participants), as previously recommended (Murias et al., 2011d). $\dot{\text{V}}$O_2_ data were modeled from the beginning of phase II to 4-min (240 s), ensuring that steady-state $\dot{\text{V}}$O_2_ had been achieved by that time. This strategy was selected so that data were fit to at least four time constants (>98% of the total response) during the on-transient of the response, avoiding superfluous sections of the steady-state data and thus maximizing the quality of the fit during the exercise on-transient. The model parameters were estimated by least-squares nonlinear regression (Origin, OriginLab Corp., Northampton, MA, USA) in which the best fit was defined by minimization of the residual sum of squares and minimal variation of residuals around the Y-axis (Y = 0). The 95% confidence interval for the estimated time constant was determined after preliminary fit of the data with Y_bsln_, Amp, and TD constrained to the best-fit values and the τ allowed to vary.

The NIRS-derived deoxy-[Hb+Mb] data were time aligned and averaged to 5-s bins to yield a single response for each participant at each of the testing points. The deoxy-[Hb+Mb] profile is described to consist of a time delay at the onset of exercise (referred to as the calculated time delay), followed by an increase in the signal with an “exponential-like” time-course (Murias et al., 2011c). The calculated time delay for the deoxy-[Hb+Mb] response (deoxy-[Hb+Mb]_CTD_) was determined using second-by-second data and corresponded to the time, after the onset of exercise, at which the deoxy-[Hb+Mb] signal began a systematic increase from its nadir value and was determined by calculating the deoxy-[Hb+Mb]_CTD_ from the averaged response of the three trials. The deoxy-[Hb+Mb] data were modeled using Equation (1); the fitting window for the “exponential” response spanned from the end of the deoxy-[Hb+Mb]_CTD_ to 90 s into the transition. As described previously (duManoir et al., 2010), different fitting strategies ranging from 90 to 180 s into a transition resulted in minimal differences in estimates of τ deoxy-[Hb+Mb]. Whereas the τ deoxy-[Hb+Mb] described the time course for the increase in deoxy-[Hb+Mb], the overall time course of the deoxy-[Hb+Mb] from the onset of the step transition was described by the effective deoxy-[Hb+Mb] (τ' deoxy-[Hb+Mb] = deoxy-[Hb+Mb]_CTD_ + τ deoxy-[Hb+Mb]).

Calculations of the deoxy-[Hb+Mb]/$\dot{\text{V}}$O_2_ ratio were similar to those previously described (Murias et al., 2011e, 2012) Briefly, the second-by-second deoxy-[Hb+Mb] and $\dot{\text{V}}$O_2_ data were normalized for each participant (0–100% of the transition response). The normalized $\dot{\text{V}}$O_2_ was left shifted to account for the phase I-phase II transition (Murias et al., 2011d) so that the beginning of phase II $\dot{\text{V}}$O_2_ coincided with the onset of exercise as detailed elsewhere (Murias et al., 2011c). An overall average deoxy-[Hb+Mb]/$\dot{\text{V}}$O_2_ ratio for the adjustment period during the exercise on-transient was derived individually as the average of the ratio values from 20 to 120 s. The start point was selected to be 20 s to begin beyond the physiological deoxy-[Hb+Mb]_CTD_ derived from NIRS. An end point of 120 s was selected to ensure that the deoxy-[Hb+Mb]/$\dot{\text{V}}$O_2_ had reached a value of 1.0, indicating a steady state in the matching of this response.

Vascular reperfusion rate was evaluated as previously established (McLay et al., 2016a,b). Briefly, the NIRS-derived O_2_ saturation (StO_2_) reperfusion rate (slope 2) was quantified as the slope of the StO_2_ signal over a 10-s window period immediately following cuff release (StO_2_%·s^−1^). It should be noted that immediately after cuff release there is a linear increase in the StO_2_, followed by a brief plateau around the peak StO_2_. This linear response is sustained for >10 s which allows for an accurate slope to be easily calculated when including the initial reperfusion phase immediately following cuff release.

### Statistical analysis

Statistical analysis was performed using SPSS 24.0 software (SPSS Inc., Chicago, IL) and Microsoft Excel. All results are presented as mean ± standard deviation (*SD*). The parameter estimates for $\dot{\text{V}}$O_2_ and deoxy-[Hb+Mb], as well as the deoxy-[Hb+Mb]/$\dot{\text{V}}$O_2_ ratio and the Slope 2 StO_2_ responses were analyzed with a two-way analysis of variance (ANOVA) using aging and fitness level as independent factors. Significant main effects and interactions were analyzed using Fisher's Least Significant Difference (LSD) *post-hoc* test. Pearson product-moment correlation was used to determine the association between variables. Statistical significance was accepted at *p* < 0.05.

## Results

Participant characteristics, as well as $\dot{\text{V}}$O_2peak_ and PO_peak_ output determined from the ramp-incremental test are listed in Table 1. As expected by design, age was significantly greater in the older participants compared to younger participants in endurance trained, recreationally active and inactive groups (*p* < 0.05). The absolute $\dot{\text{V}}$O_2peak_ and PO_peak_ output were higher in younger groups compared with that of older groups across each fitness level (*p* < 0.05). Additionally, absolute $\dot{\text{V}}$O_2peak_ and PO_peak_ were lower in inactive groups than endurance trained groups in both younger and older participants (*p* < 0.05).

**Table 1:** Participant characteristics and peak exercise responses.

	**YI**	**YR**	**YT**	**OI**	**OR**	**OT**
*n*	10	10	10	7	10	10
Age (yr)	26 ± 5	26 ± 6	30 ± 5	68 ± 3^*^	68 ± 5^*^	66 ± 3^*^
Height (cm)	176 ± 6	179 ± 7	179 ± 5	171 ± 7	176 ± 5	178 ± 7
Weight (kg)	79 ± 17	80 ± 7	76 ± 7	77 ± 10	82 ± 9	77 ± 9
BMI (kg/m^2^)	25 ± 4	25 ± 2	24 ± 2	26 ± 4	27 ± 3	24 ± 2
$\dot{\text{V}}$O_2peak_ (L·min^−1^)	2.96 ± 0.55	3.92 ± 0.33^†^	4.42 ± 0.32^†^	2.50 ± 0.31^*^	2.71 ± 0.42^*^	3.20 ± 0.35^†^^,^^‡^^,^^*^
GET (L·min^−1^)	1.82 ± 0.15	2.08 ± 0.11^†^	2.57 ± 0.34^†^^,^^‡^	1.50 ± 0.21^*^	1.70 ± 0.18^†^^,^^*^	1.92 ± 0.19^†^^,^^‡^^,^^*^
PO_peak_ (W)	249 ± 40	362 ± 35^†^	420 ± 37^†^^,^^‡^	237 ± 21^*^	256 ± 32^*^	303 ± 29^†^^,^^‡^^,^^*^

Values are means ± SD. BMI, body mass index; $\dot{\text{V}}$O_2peak_, peak oxygen uptake; GET, gas exchange threshold; PO_peak_, peak power output.

† Significantly different from inactive group (p < 0.05).

‡ Significantly different from recreationally active group (p < 0.05).

* *Significantly different from training-matched age group (p < 0.05)*.

### $\dot{\text{V}}$O_2_ and Deoxy-[Hb+Mb] kinetics

**Figure 1:**
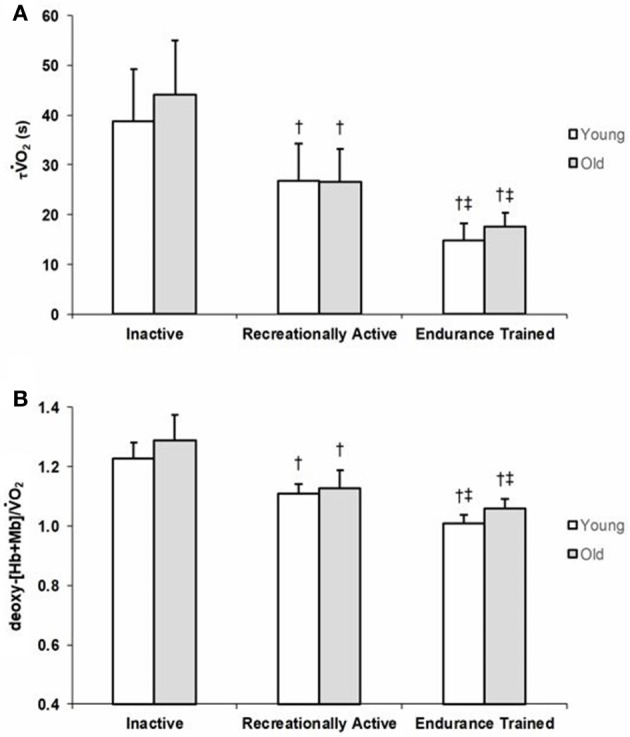
Group mean τ$\dot{\text{V}}$O_2_ **(A)** and deoxy-[Hb+Mb]/$\dot{\text{V}}$O_2_ ratio **(B)** values for young and older inactive (I), recreationally active (R), and endurance trained (T) groups. Data are presented as mean ± *SD*. Significantly different from inactive group (*p* < 0.05). ^‡^Significantly different from recreationally active group (*p* < 0.05).

**Figure 2:**
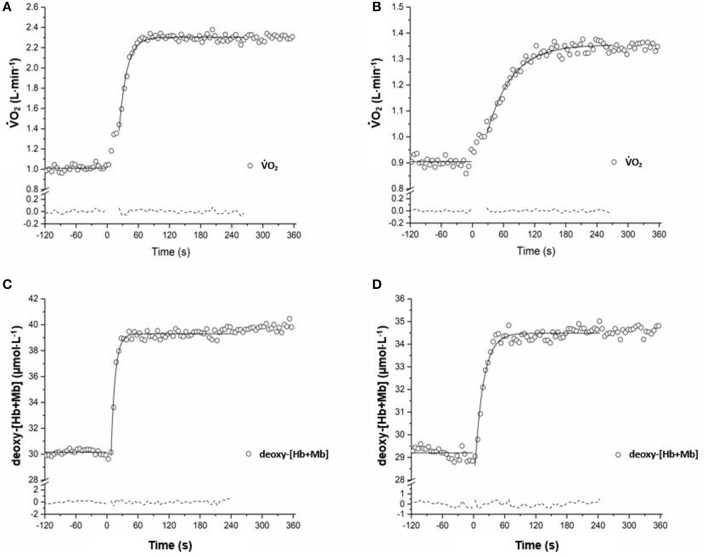
Adaptation of $\dot{\text{V}}$O_2_ **(A,B)** and *vastus lateralis* deoxygenation **(C,D)** during the transition to exercise at a relative WR corresponding to ~80% of the gas exchange threshold in a representative YT and OI adult. Model fits of the data are displayed with a *solid line*, and residuals of the fit are displayed with a *dashed line*.

Group means and *SD* for τ $\dot{\text{V}}$O_2_ data are displayed in Figure 1A and detailed in Table 2. Figures 2 A,B show a representative fit for the $\dot{\text{V}}$O_2_ kinetics in a young and older participant, respectively. The τ$\dot{\text{V}}$O_2_ was greater in the: (1) inactive groups when compared to the recreationally active or endurance trained groups (*p* < 0.05), and (2) recreationally active groups compared to the endurance trained groups (*p* < 0.05). There was no main effect for aging or aging by fitness level interaction for τ$\dot{\text{V}}$O_2_ (*p* > 0.05). Due to the differences in absolute WR during MOD, $\dot{\text{V}}$O_2_ amplitude was significantly lower in older recreationally trained and endurance trained individuals than their young training-matched counterparts (*p* < 0.05); however, there was no difference between young inactive and older inactive groups (*p* > 0.05). While, $\dot{\text{V}}$O_2_ amplitude and MOD WR were greater in older endurance trained individuals compared to both inactive and recreationally active (*p* < 0.05), there was a progressive increase in the $\dot{\text{V}}$O_2_ amplitude and MOD WR corresponding to any increase in fitness level in the younger groups (*p* < 0.05) (Table 2). The $\dot{\text{V}}$O_2_ baseline was greater in younger compared to older individuals (*p* < 0.05), however, the functional $\dot{\text{V}}$O_2_ gain (Δ$\dot{\text{V}}$O_2_/ΔWR) was not different in any of the groups (*p* > 0.05) (Table 2). τ deoxy-[Hb+Mb], CTD deoxy-[Hb+Mb] and τ' deoxy-[Hb+Mb] were not statistically different across all aging and training groups (*p* > 0.05) (Table 2). Figures 2C,D shows a representative fit for the deoxy-[Hb+Mb] kinetics in a young and in an older participant, respectively. There was a significant negative correlation between $\dot{\text{V}}$O_2peak_ and τ$\dot{\text{V}}$O_2_ in both older (*r* = 0.60; *p* < 0.05) and young (*r* = 0.63; *p* < 0.05) participants.

**Table 2:** $\dot{\text{V}}$O_2_ and deoxy-[Hb+Mb] kinetic parameters for the transition to moderate intensity exercise.

	**YI**	**YR**	**YT**	**OI**	**OR**	**OT**
**$\dot{\text{V}}$O_2_ KINETICS**						
$\dot{\text{V}}$O_2__BSLN_ (L·min^−1^)	1.15 ± 0.15	1.13 ± 0.13	1.02 ± 0.09^§^^,^^Φ^	0.90 ± 0.15^*^	1.06 ± 0.08^*^	1.09 ± 0.09^§^^,^^*^
$\dot{\text{V}}$O_2__AMP_ (L·min^−1^)	0.51 ± 0.13	0.77 ± 0.15^†^	1.22 ± 0.30^†^^,^^‡^	0.45 ± 0.13^*^	0.45 ± 0.12^*^	0.58 ± 0.11^†^^,^^‡^^,^^*^
$\dot{\text{V}}$O_2__GAIN_ (mL·W^−1^)	10.3 ± 0.5	10.1 ± 0.5	10.4 ± 1.4	10.2 ± 1.7	10.0 ± 0.7	10.4 ± 0.7
TD $\dot{\text{V}}$O_2_ (s)	14.2 ± 6.9	13.2 ± 4.1	16.7 ± 1.7	15.1 ± 6.1^*^	18.6 ± 5.9^*^	20.4 ± 3.1^*^
τ$\dot{\text{V}}$O_2_ (s)	38.8 ± 10.4	26.8 ± 7.5^†^	15.0 ± 3.4^†^^,^^‡^	44.8 ± 10.9	26.6 ± 6.5^†^	16.9 ± 2.7^†^^,^^‡^
CI_95_ (s)	7.8 ± 2.5	4.3 ± 1.2^†^	2.0 ± 0.9^†^^,^^‡^	7.7 ± 5.5^*^	6.3 ± 2.6^†^^*^	4.1 ± 1.0^†^^,^^‡^^,^^*^
**Deoxy-[Hb+Mb] KINETICS**						
CTD deoxy-[Hb+Mb] (s)	8.9 ± 3.6	10.0 ± 1.8	8.0 ± 2.4	12.2 ± 5.0	9.2 ± 2.6	10.4 ± 3.9
τ deoxy-[Hb+Mb] (s)	12.5 ± 3.1	9.0 ± 2.9	7.2 ± 3.4	9.2 ± 1.8	13.4 ± 7.5	10.1 ± 4.4
τ' deoxy-[Hb+Mb] (s)	21.4 ± 4.6	19.0 ± 3.1	15.2 ± 3.9	21.4 ± 4.5	22.6 ± 8.6	20.5 ± 2.7
deoxy-[Hb+Mb]/$\dot{\text{V}}$O_2_	1.23 ± 0.05	1.11 ± 0.03^†^	1.01 ± 0.03^†^^,^^‡^	1.29 ± 0.08	1.13 ± 0.06^†^	1.06 ± 0.03^†^^,^^‡^
MOD (W)	69 ± 12	96 ± 14^†^	137 ± 24^†^^,^^‡^	64 ± 13	65 ± 12^*^	76 ± 10^†^^,^^‡^^,^^*^

Values are mean ± SD. τ, time constant of response; τ' deoxy-[Hb+Mb], sum of τ deoxy-[Hb+Mb] and CTD deoxy-[Hb+Mb]; BSLN, baseline; AMP, Amplitude; GAIN, functional gain; MOD, moderate intensity work rate; CI_95_, 95% confidence interval; CTD, calculated time delay for deoxy-[Hb+Mb]; deoxy-[Hb+Mb], deoxygenated hemoglobin concentration; TD, time delay.

† Significantly different from inactive group (p < 0.05);

‡ Significantly different from recreationally active group (p < 0.05);

* Significantly different from training-matched age group (p < 0.05);

§ Significantly different from age-matched inactive group (p < 0.05);

Φ *Significantly different from age-matched recreationally active group (p < 0.05)*.

### Deoxy-[Hb+Mb]/$\dot{\text{V}}$O_2_ ratio

Average group data for the deoxy-[Hb+Mb]/$\dot{\text{V}}$O_2_ ratio derived from the normalized responses of deoxy-[Hb+Mb] and $\dot{\text{V}}$O_2_ adjustments to a moderate-intensity WR are portrayed in Figure 1B. The deoxy-[Hb+Mb]/$\dot{\text{V}}$O_2_ ratio was significantly greater in both OI and YI compared to OR and YR as well as OT and YT (*p* < 0.05) (Table 2). Additionally, OR and YR displayed a significantly greater ratio than OT and YT (*p* < 0.05) (Table 2). There was a main effect for aging (*p* < 0.05) and no aging by training interaction for the deoxy-[Hb+Mb]/$\dot{\text{V}}$O_2_ ratio (*p* > 0.05).

### NIRS-derived slope 2 of StO_2_

**Figure 3:**
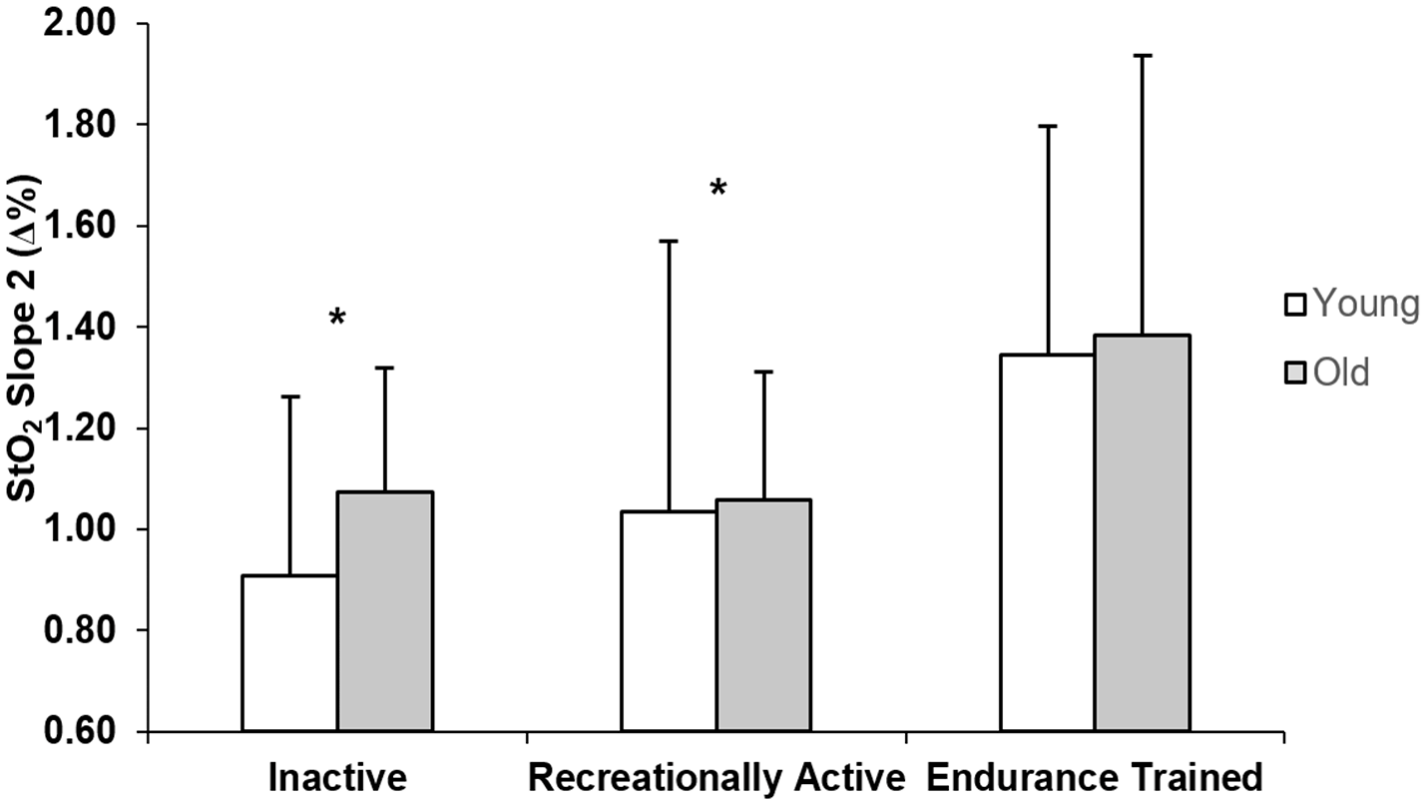
Group mean NIRS-derived StO_2_ values of slope 2 for young and older inactive (I), recreationally active (R), and endurance trained (T) groups. *Significantly different from endurance trained group (*p* < 0.05).

Figure 3 displays the Slope 2 StO_2_ values for young and older inactive, recreationally active, and endurance trained individuals. There was a main effect of training (*p* = 0.033), whereby inactive (*p* = 0.018) and recreationally active men (*p* = 0.031) had signifcantly lower values of slope 2 than endurance trained men regardless of age.

## Discussion

The present study examined the effects of aging and fitness level on the $\dot{\text{V}}$O_2_ kinetics profiles of young and older inactive, recreationally active, and endurance trained men. The main findings are as follows. Firstly, across all training groups, $\dot{\text{V}}$O_2_ kinetics were not slower in older adults compared to their training-matched younger counterparts. This study is the first to show that fitness level and not aging *per se* determines the $\dot{\text{V}}$O_2_ kinetics response across a wide range of training levels. Secondly, the present data indicate that, along with other intracellular mechanisms of control that were not measured in the present study, improved matching of O_2_ delivery to O_2_ utilization seems to be an important factor in determining the speed of the $\dot{\text{V}}$O_2_ kinetics response, across all aging and training groups.

Studies assessing the $\dot{\text{V}}$O_2_ kinetics response in young and older adults have regularly shown slower τ$\dot{\text{V}}$O_2_ values in the aging population (Chilibeck et al., 1996; DeLorey et al., 2004; Murias et al., 2010; Gravelle et al., 2012). Although debate exists in relation to the mechanisms that control the rate of adjustment of oxidative phosphorylation (Grassi, 2001; Hughson et al., 2001; Poole and Jones, 2012; Murias et al., 2014; Murias and Paterson, 2015), there is a stronger consensus supporting the idea that, in older populations, the slower dynamic adjustment of $\dot{\text{V}}$O_2_ is related to limitations within the O_2_ transport system and, more specifically, in the O_2_ provision to the active muscles which affects the matching of O_2_ delivery to O_2_ utilization (Murias et al., 2010; Poole and Jones, 2012). However, a slower adjustment of oxidative phosphorylation in older individuals is not always the case. In a review by Murias and Paterson (2015), the authors presented the τ$\dot{\text{V}}$O_2_ values of older (*n* = 41) and younger (*n* = 49) individuals demonstrating a large overlap of τ$\dot{\text{V}}$O_2_ values between age groups. These data denote that while a random sample of older individuals is likely to display a slower $\dot{\text{V}}$O_2_ kinetics, they can also have a τ$\dot{\text{V}}$O_2_ just as fast as a young individual. In fact, some short-term (≤12 weeks) training studies have demonstrated an acceleration of the $\dot{\text{V}}$O_2_ kinetics response from ~40–45 to ~30–35 s in older individuals (Fukuoka et al., 2002; Murias et al., 2010), and from ~30–35 to ~20–25 s in young individuals (Berger et al., 2006; Murias et al., 2010). Furthermore, Grey et al. (2015) showed that older, endurance-trained individuals had a $\dot{\text{V}}$O_2_ kinetics response as rapid as their younger training-matched counterparts (~18 s) which aligns with the results of the current investigation (~16 s τ$\dot{\text{V}}$O_2_ values). Importantly, this study fills a gap in the literature, as it includes young and older participants from across a wide training spectrum. One earlier study assessed $\dot{\text{V}}$O_2_ kinetics across the aging spectrum (i.e., young, middle-aged, and older men) in chronically trained and healthy active community dwelling individuals (Grey et al., 2015), however it was limited by the lack of an inactive group. This is an important component since, if fitness level and not aging *per se* is the main factor modulating the speed of the $\dot{\text{V}}$O_2_ kinetics response, then it would be expected that young inactive men would have a τ$\dot{\text{V}}$O_2_ that is not different from that observed in their inactive older counterparts. This study is the first to demonstrate that regardless of age, τ$\dot{\text{V}}$O_2_ is affected by fitness level in a group of young and older individuals that spanned over a wide range of training levels (Figure 1A, Table 2). Most notably, group mean τ$\dot{\text{V}}$O_2_ of young inactive adults was not different from that of older inactive males, emphasizing that it is not only the accumulation of many years of an inactive lifestyle that produce detrimental cardiovascular adaptations affecting the O_2_ transport system, but that these negative effects can be observed early in the life span when inactive behaviors are adopted. Further support to the connection between fitness status and the dynamic adjustment of $\dot{\text{V}}$O_2_ comes from the large correlation between $\dot{\text{V}}$O_2max_ and τ$\dot{\text{V}}$O_2_.

The mechanisms responsible for the rate of adjustment of $\dot{\text{V}}$O_2_ have been a matter of debate for years. Although intracellular mechanisms of control are undeniably an important component controlling the dynamic adjustment of oxidative phosphorylation during exercise transitions to higher metabolic demands (Grassi, 2000; Grassi et al., 2002; Rossiter, 2010; Poole and Jones, 2012), several recent studies (Murias et al., 2010; Grey et al., 2015; McLay et al., 2017) have indicated that adequate provision of O_2_ to the active tissues is also a key rate limiting mechanism. In relation to the latter, the present study reports that the matching of O_2_ delivery to O_2_ utilization in the active muscles, as indicated by the deoxy-[Hb+Mb]/$\dot{\text{V}}$O_2_ ratio, is improved in recreationally active and endurance trained groups compared to inactive groups, and further improved in endurance trained groups compared to recreationally active groups (Figure 1, Table 2). Further support is thus provided for these studies that have proposed increased O_2_ distribution to the sites of increased metabolic demand playing an important role in the regulation of $\dot{\text{V}}$O_2_ during exercise (Murias et al., 2010, 2011a, 2014; McLay et al., 2016a). For example, improved matching of O_2_ delivery to O_2_ utilization, which was associated to a faster $\dot{\text{V}}$O_2_ kinetics response, has been demonstrated following brief (≤3 sessions) (Murias et al., 2016; McLay et al., 2017), short-term (~12 weeks) (Murias et al., 2010, 2011a) and long-term (Grey et al., 2015) training in both young and older participants. Although not investigated in this study, improved matching of O_2_ delivery to O_2_ utilization within the active tissues can be explained by different structural enhancements related to exercise training. For example, Behnke et al. (2012), demonstrated an increase in the cross-sectional area (CSA) of feed arteries to the muscle after training in old rats. This increased CSA was responsible for increased blood flow and improved matching of O_2_ delivery to O_2_ consumption. Similarly, Spier et al. (2007) indicated that exercise training restores flow-mediated vasodilatory responses in gastrocnemius muscle arterioles from old rats. Similarly, improved vascular responsiveness through enhanced endothelium dependent vasodilation has been indicated to occur in response to exercise training (DeSouza et al., 2000). Additionally, although the role of the capillaries for blood-myocyte O_2_ exchange has been questioned (Poole et al., 2013), improvements in the potential for O_2_ flux from capillaries to muscles fibers can be observed in chronically trained masters athletes who demonstrated similar capillary density to training-matched young athletes (Coggan et al., 1990), or by increases in measures of capillarization that take place in response to endurance training interventions (Coggan et al., 1992; Murias et al., 2011b).

Further support for this theory is provided through the results of the NIRS-derived measure of microvascular reperfusion presented in this study. There was a significant main effect of training for the Slope 2 StO_2_, that demonstrated not only improved vascular responsiveness in the endurance trained compared to the recreationally active and inactive individuals, but also a similar effect of training independently of the age group (Figure 3). Murias et al. (2010, 2011a) previously proposed that improved vascular responsiveness resulted in more adequate matching of O_2_ delivery to O_2_ utilization within the active tissues, which in turn played a role for speeding of the $\dot{\text{V}}$O_2_ kinetics response. That suggestion was further supported by a study showing that speeding and slowing of $\dot{\text{V}}$O_2_ kinetics were accompanied by decreases and increases in the deoxy-[Hb+Mb]/$\dot{\text{V}}$O_2_ ratio, as well as increased and decreased measures of flow mediated dilation (FMD), respectively. A novel aspect related to this study is the examination the differences in the Slope 2 StO_2_, which estimates vascular responsiveness within the microcirculation (near the tissues in which changes in $\dot{\text{V}}$O_2_ occur), rather than in a conduit artery as FMD (McLay et al., 2016a). The steeper slopes observed in the endurance trained group might be due to long-term endurance training-stimulated vascular remodeling that the muscles undergo to provide a system that is capable of properly matching the O_2_ delivery to the metabolic requirements of the fibers, at least during exercises performed in the moderate intensity domain. A limitation of the Slope 2 StO_2_ analysis is that, even though it has been indicated to show less variability than measures of FMD, ~25–30% of the response might be affected by variability (McLay et al., 2016b), making the dispersion of this response larger, and differences less noticeable in the groups with lower fitness level.

Although our data supports the idea that an O_2_ delivery limitation might explain part of the slower $\dot{\text{V}}$O_2_ kinetics observed in the individual with progressively lower fitness level, an alternative hypothesis suggests that the main mechanisms controlling the dynamic adjustment of oxidative phosphorylation reside intracellularly, and thus changes related to training are explained by intracellular adaptations that improves provision of substrates, other than O_2_, to the mitochondria (Grassi et al., 1998a,b; Poole and Jones, 2012). The use of 31-phosphorous magnetic resonance spectroscopy (^31^P-MRS) to infer the adjustment of muscle $\dot{\text{V}}$O_2_ by measuring PCr breakdown (McCreary et al., 1996) has been used by many research groups to support a theory of intracellular control (Barstow et al., 1994a,b; McCreary et al., 1996; Rossiter et al., 1999, 2003). Additionally, pyruvate dehydrogenase (PDH) has been investigated as a potential site of regulation for oxidative phosphorylation (Howlett et al., 1999; Bangsbo et al., 2002; Grassi et al., 2002; Rossiter et al., 2003; Jones et al., 2004). However, to date, experiments in humans (Bangsbo et al., 2002; Rossiter et al., 2003; Jones et al., 2004) have failed to demonstrate faster $\dot{\text{V}}$O_2_ kinetics following prior PDH activation via dichloroacetate supplementation. In relation to exercise training, one study has proposed that the speeding of the $\dot{\text{V}}$O_2_ kinetics response following a 20-week endurance training protocol was mainly due to an increase in each-step parallel activation rather than mitochondrial biogenesis (Zoladz et al., 2014). An important observation to make about that study is that changes in the $\dot{\text{V}}$O_2_ kinetics response were measured 20 weeks apart, and several investigations have shown that changes in the adjustment of the $\dot{\text{V}}$O_2_ kinetics occur within a few training sessions (Murias et al., 2016; McLay et al., 2017). Thus, there is a disconnection between the time of measurement and the time at which the changes occurred. Nevertheless, it has to be acknowledged that a large part of the literature supports the idea that intracellular mechanisms of control are the key regulators of the $\dot{\text{V}}$O_2_ kinetics response and that, consequently to this idea, exercise training not only results in improvements in vascular responsiveness and blood flow distribution, but also upregulate intracellular pathways linked to oxidative metabolism (Henriksson and Reitman, 1977; Örlander and Aniansson, 1980; Coggan et al., 1990, 1992; Belman and Gaesser, 1991; Murias et al., 2011b). Thus, the present study did not aim to isolate the mechanisms that control the dynamic adjustment of $\dot{\text{V}}$O_2_. Importantly, what this study highlights is that, independently of what the mechanisms of control are, the $\dot{\text{V}}$O_2_ kinetics response is linked to fitness status and that this link is not affected by the age of the participants (i.e., not only that older trained individuals might display $\dot{\text{V}}$O_2_ kinetics as fast as those observed in their younger counterparts, but also that inactive but otherwise healthy young individuals might have a $\dot{\text{V}}$O_2_ kinetics response that is as slow as that observed in their older counterparts).

Two different factors could be considered as limitations in this study: (1) although this study indicates that there is a connection between fitness status and the $\dot{\text{V}}$O_2_ kinetics response, it could be argued that the strong negative correlation between $\dot{\text{V}}$O_2peak_ and τ$\dot{\text{V}}$O_2_ is not that close to a perfect correlation. However, it should be noted that it is not the $\dot{\text{V}}$O_2peak_ *per se*, but what having a high $\dot{\text{V}}$O_2peak_ represents from a cardiovascular and metabolic adaptions perspective what modulates the $\dot{\text{V}}$O_2p_ kinetics response. For example, a chronically trained older person would have a lower maximal heart rate, which will reduce maximal cardiac output, and thus $\dot{\text{V}}$O_2peak_. However, this same older person might still benefit from most of the other cardiovascular and metabolic adaptations as expected in a young trained individual. All these beneficial training adaptations will result in a very fast $\dot{\text{V}}$O_2_ kinetics response during submaximal tasks as measured in this study. Thus, the aspect that will limit $\dot{\text{V}}$O_2peak_ in this example, should not impair the adjustment of the $\dot{\text{V}}$O_2_ kinetics response; (2) the adjustment of the deoxy-[Hb+Mb] was only tested in the vastus lateralis muscle. Different lines of research have indicated heterogeneous responses between and within the muscle in the amplitude and dynamic profiles of tissue oxygenation signals (Koga et al., 2007; Heinonen et al., 2015; Iannetta et al., 2017). Therefore, data from the vastus lateralis muscle should not be interpreted as a representation of the activity of all the muscles involved in cycling.

In conclusion, this study demonstrated not only that age-related slowing of $\dot{\text{V}}$O_2_ kinetics can be eliminated when endurance training exercise has been performed, but also that an inactive lifestyle has a negative impact on the $\dot{\text{V}}$O_2_ response of young and otherwise healthy individuals. Unique to this study was the lack of statistical differences between the τ$\dot{\text{V}}$O_2_ values and deoxy-[Hb+Mb]/$\dot{\text{V}}$O_2_ ratios of every training group, regardless of the age of the participants. These data indicate that fitness level and not aging *per se* determine the $\dot{\text{V}}$O_2_ kinetics response and further highlight the importance of endurance training to improve or sustain cardiovascular function.

## Author contributions

MG: Participated in the design, recruited participants, collected and analyzed data, and prepared the manuscript for submission; KM: Assisted with data collection and design; RR and PD-B: Contributed to the design of the project and provided feedback on the manuscript; JM: Contributed to designing the project, analyzing the data, and writing the manuscript.

### Conflict of interest statement

The authors declare that the research was conducted in the absence of any commercial or financial relationships that could be construed as a potential conflict of interest.
